# Green vessel signal on multicolor imaging as an early sign of ischemic vasculitis in IRVAN Syndrome: A case report

**DOI:** 10.1016/j.ajoc.2026.102544

**Published:** 2026-02-12

**Authors:** Daniel M. Markowitz, Jirat Nimworaphan, Carl D. Regillo, James P. Dunn, Robert C. Sergott

**Affiliations:** a Drexel University College of Medicine, Philadelphia, PA, USA; b William H. Annesley Jr. EyeBrain Center, Vicky and Jack Farber Neuroscience Institute, Thomas Jefferson University, Partnered with Wills Eye Hospital, Philadelphia, PA, USA; c Faculty of Medicine Vajira Hospital, Department of Ophthalmology, Navamindradhiraj University, Bangkok, Thailand; d Retina Service, Mid Atlantic Retina, Wills Eye Hospital, Thomas Jefferson University, Philadelphia, PA, USA

## Abstract

**Purpose:**

Idiopathic retinitis, vasculitis, aneurysms, and neuroretinitis (IRVAN) syndrome is typically diagnosed after characteristic intravenous fluorescein angiography (IVFA) findings develop. This case demonstrates that identifying a green intravascular and perivascular signal on multicolor fundus imaging may be a potential noninvasive imaging sign for ischemic retinal vasculitis in IRVAN.

**Observations:**

A 55-year-old female presented with bilateral optic disc edema and subtle peripapillary retinal vascular changes. Multicolor imaging showed a distinct green intravascular and perivascular signal before undergoing IVFA evaluation. Subsequent IVFA ultimately showed peripheral capillary nonperfusion, microaneurysms, and leakage consistent with IRVAN.

**Conclusions and importance:**

This case demonstrates the potential utility of multicolor imaging for detecting subtle vascular abnormalities that are not apparent on clinical examination or on traditional color fundus photography. Recognition of this green vessel signal may serve as an imaging sign of vascular wall changes, prompting consideration of IRVAN and guiding timely further angiographic evaluation in patients presenting with atypical optic disc edema or suspected retinal vasculitis.

## 1 Introduction

Idiopathic retinitis, vasculitis, aneurysms, and neuroretinitis (IRVAN) syndrome is characterized by arterial aneurysms, peripheral capillary nonperfusion, and neuroretinitis.^1^^,^^2^ Its underlying pathophysiology remains uncertain, although inflammatory mechanisms are suggested based on intravenous fluorescein angiography (IVFA) findings and reported therapeutic responses to immunomodulatory medications.^3^^,^^4^ Developmental vascular anomalies have also been proposed as contributing factors.^5^

Early diagnosis can be challenging because patients may present with isolated optic disc edema or subtle fundoscopic changes, leading to a broad differential diagnosis. In these settings, multimodal imaging plays an important role in characterizing retinal vascular and inflammatory changes that may not be apparent clinically.

This case describes a patient with IRVAN syndrome in which multicolor imaging revealed subtle vascular abnormalities during the early course of disease, preceding subsequent evaluation with IVFA. Multicolor imaging may complement existing diagnostic modalities in the initial evaluation of atypical papillitis and retinal vascular disease.

## 2 Case presentation

A 55-year-old female presented with several months of progressive blurred vision in both eyes, intermittent headaches, and new floaters. Visual acuity was 20/30 in both eyes (OU), with no improvement with pinholes of the right eye (OD), mild improvement with pinholes to 20/25 of the left eye (OS), and normal intraocular pressures of 14 mmHg OU. Color plates were 8/8 bilaterally, without an afferent pupillary defect, and the anterior segment exam was normal.

**Fig. 1:**
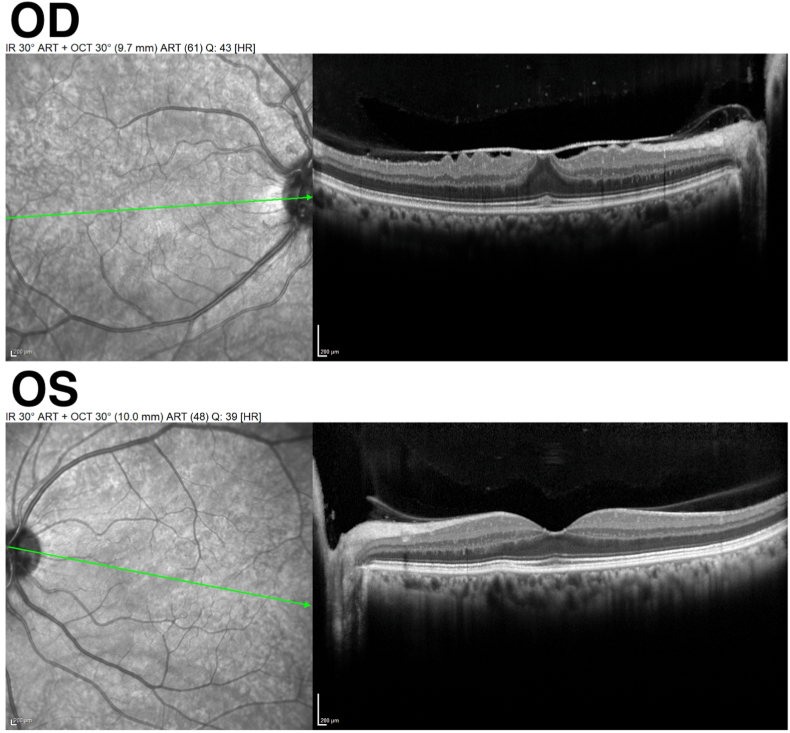
OCT line scans of the macula in both eyes. The right eye has a mild epiretinal membrane, causing traction and disruption of the foveal contour but without edema. The left eye shows a normal foveal contour and is without macular edema.

Fundus examination showed 1+ anterior vitreous cells and mild bilateral optic disc edema, more pronounced in the right eye. Spectral domain optical coherence tomography (OCT) of the macula demonstrated a mild epiretinal membrane in the right eye (Fig. 1). Peripapillary retinal nerve fiber layer thickness was 117 μm OD and 101 μm OS.

**Fig. 2:**
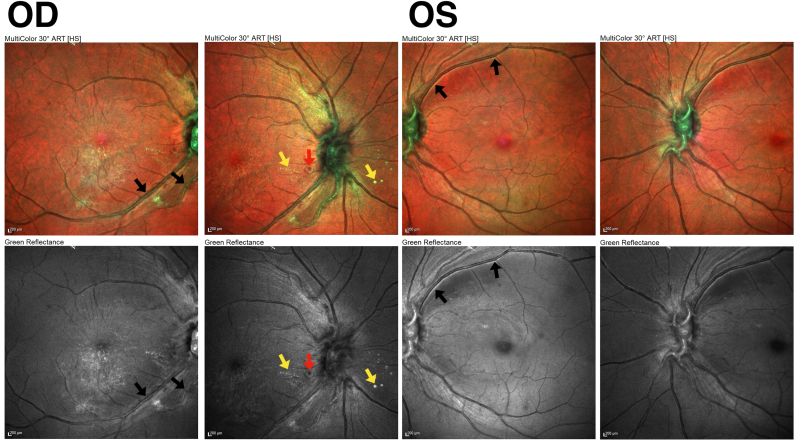
Composite multicolor images (top row) and green reflectance images (bottom row) of the right and left eyes show green intravascular and perivascular signal along the retinal arterioles, suggestive of early vascular wall change (black arrows). The right eye shows peripapillary hemorrhage (red arrow) and exudate (yellow arrows). (For interpretation of the references to color in this figure legend, the reader is referred to the Web version of this article.)

Multicolor imaging (Spectralis Multicolor Module, Heidelberg Engineering) identified peripapillary hemorrhages and exudate in the right eye and a green intravascular and perivascular signal in both eyes on the composite image, and as a white signal on the green reflectance image, raising suspicion for an underlying vascular ischemic process (Fig. 2).

Initial laboratory evaluation, including complete blood count, antinuclear antibody, cytoplasmic and perinuclear antineutrophil cytoplasmic antibody, C-reactive protein, Quantiferon gold, Lyme testing, and hemoglobin A1C was unremarkable. MRI of the brain and orbits showed only mild nonspecific cerebral white matter changes without enhancement of any region of the afferent visual system.

After an unremarkable systemic and neuroimaging workup, the etiology of the optic disc edema remained unclear. Given the absence of clinical or ancillary findings suggestive of a classic retinal vasculitis, the patient was monitored clinically. Despite stable visual acuity, she continued to report intermittent headaches, and follow-up imaging one year later revealed new peripheral vascular changes that prompted IVFA evaluation.

**Fig. 3:**
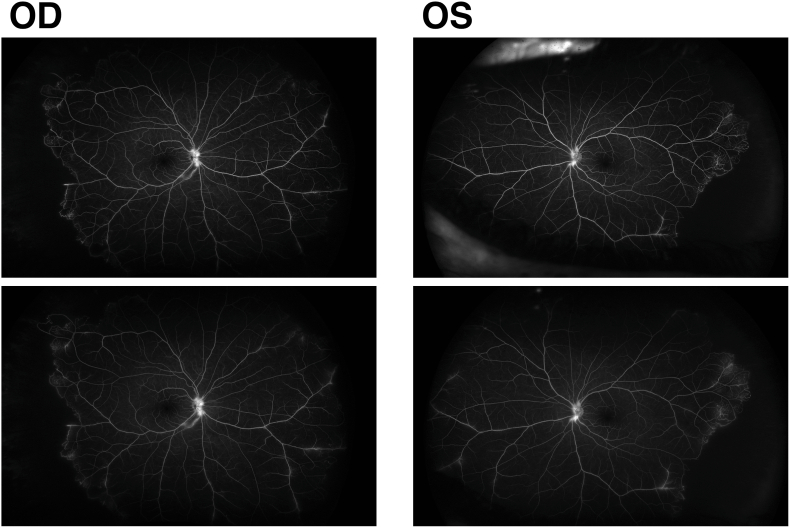
Fluorescein angiography of both eyes in the venous (top row) and late venous phases (bottom row) shows optic disc hyperfluorescence, peripheral capillary nonperfusion with microaneurysms, and late venous leakage. Mild proximal arterial leakage is noted in the right eye.

**Fig. 4:**
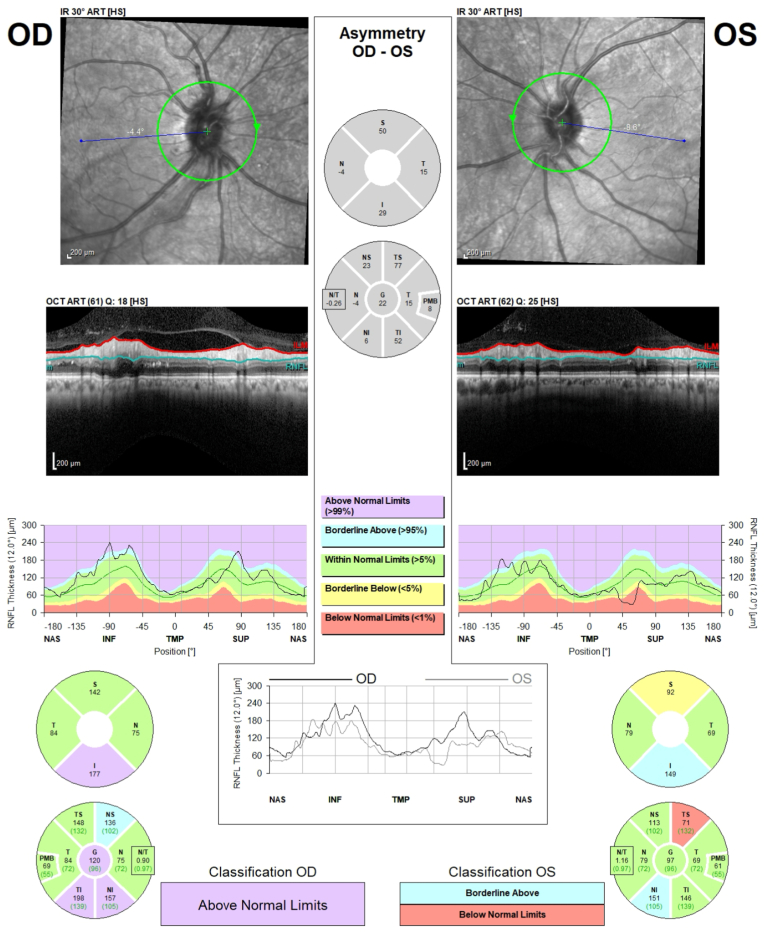
OCT of the peripapillary retinal nerve fiber layer shows mild thickening in both eyes, more in the right. Retinal nerve fiber layer thickness was 120 μm OD and 97 μm OS.

Widefield pseudo-color fundus photos showed proximal arterial and venous sheathing and peripheral intraretinal hemorrhages in both eyes. IVFA demonstrated peripheral capillary nonperfusion, microaneurysms, and both arterial and venous leakage in both eyes, consistent with IRVAN syndrome (Fig. 3). At that time, peripapillary RNFL thickness was 120 μm OD and 97 μm OS (Fig. 4).

Panretinal photocoagulation (PRP) was performed in both eyes. At follow-up 14 months after PRP, optic disc edema had improved, visual acuity was stable at 20/25 OU, and IVFA showed reduced leakage. Persistent peripheral retinal ischemia, mild RNFL thickening, and green vessel signal on multicolor imaging remained stable on subsequent examinations.

## 3 Discussion

IRVAN syndrome is usually diagnosed once aneurysms, peripheral capillary nonperfusion, and neuroretinitis become apparent on IVFA. Although the evolution of IRVAN features can be variable, many cases present with emerging aneurysms, capillary nonperfusion, and neuroretinitis, sometimes with significant peripheral ischemia at the time of diagnosis.^2^ In contrast, our patient initially demonstrated optic disc edema and subtle vascular changes on multicolor imaging, leading to diagnostic uncertainty during the early phase of her presentation.

Multicolor imaging in this case revealed a green intravascular and perivascular signal prior to definitive IVFA. In contrast to Lin et al., where multicolor imaging primarily showed optic disc or surface retinal changes, our case shows that multicolor imaging can highlight subtle vascular wall abnormalities earlier in the disease course.^6^ While the underlying pathophysiologic mechanism generating this green signal is not fully established, it may indicate blood vessel wall breakdown or surrounding edema related to ischemia or inflammation.^7^ Prior studies of multicolor imaging in diabetic retinopathy and retinal vascular occlusions have shown that retinal vessel wall changes and inner retinal edema may appear as a green signal on the multicolor composite image and as a white signal on the green reflectance image 8, 9, 10, 11. Our case extends these observations by suggesting that similar multicolor imaging findings may be detectable in early IRVAN and may accentuate early retinal vascular changes that are less apparent on standard white-light color fundus images.^9^ For this patient, the green signal identified on multicolor imaging raised suspicion for an underlying vascular ischemic and/or inflammatory process when fundus examination only revealed optic disc edema.

At the patient's initial presentation, IVFA was not obtained because neither her clinical examination nor ancillary testing demonstrated features suggestive of a classic retinal vasculitis. The patient was monitored over time, and as her symptoms persisted and peripheral retinal changes became more apparent, IVFA performed at follow-up confirmed the characteristic ischemia and retinal findings consistent with IRVAN. The progressive timeline in this case suggests that multicolor imaging may reveal subtle vascular changes that could raise suspicion for IRVAN or other ischemic vasculitides, even when classic angiographic features have not yet fully developed, but does not replace IVFA confirmation. However, these findings should be interpreted with caution, as multicolor imaging is limited in that it does not assess retinal perfusion, as IVFA does, which is crucial for diagnosis.

Compared with previously reported IRVAN case reports, our patient had a slower time course to the development of the classic findings seen in IVFA for IRVAN. The progression of IRVAN is unpredictable, with some cases describing rapid progression to extensive ischemia, while others describe a slower course with limited early fundus examination findings.^12^ The detection of a green signal, suggestive of vascular wall changes, on multicolor before overt IVFA changes may help ophthalmologists consider IRVAN as part of the differential diagnosis of atypical papillitis or optic disc edema and prompt further angiographic evaluation. While IVFA remains the gold standard for diagnosing IRVAN, the quick, noninvasive methodology of multicolor imaging could serve as a first step in the workup of atypical cases of papillitis or optic disc edema.

This case has several limitations. As a single case report, the observations regarding multicolor imaging in IRVAN cannot be generalized without the inclusion of larger cohorts of IRVAN patients and further study. Additionally, the prolonged interval between the patient's initial presentation and the eventual diagnosis confirmed by IVFA limits our ability to determine the precise stage at which the multicolor abnormalities first became detectable. Another limitation is that adjacent peripapillary hemorrhages may have partially influenced the green signal observed on multicolor imaging and should therefore be interpreted with caution when assessing for early ischemic or inflammatory vascular changes. Although the patient demonstrated clinical stability following PRP, long-term outcomes remain unknown. Future studies comparing multicolor imaging with IVFA and OCT angiography in larger IRVAN cohorts may clarify the sensitivity and specificity of early multicolor imaging findings.

In summary, this case illustrates that green intravascular and perivascular signal on multicolor imaging may represent a noninvasive imaging sign of ischemic retinal vasculitis in IRVAN, enabling more precise diagnostic evaluation with IVFA. Recognition of this signal should prompt ophthalmologists to consider IRVAN in the differential diagnosis of atypical papillitis and to pursue confirmatory IVFA when appropriate. While it cannot replace IVFA, multicolor imaging serves as a practical first step in identifying vascular involvement, helping to guide diagnostic investigation in evolving cases.

## CRediT authorship contribution statement

**Daniel M. Markowitz:** Writing – review & editing, Writing – original draft, Investigation, Conceptualization. **Jirat Nimworaphan:** Writing – review & editing, Writing – original draft, Investigation, Conceptualization. **Carl D. Regillo:** Writing – review & editing, Investigation. **James P. Dunn:** Writing – review & editing, Investigation. **Robert C. Sergott:** Writing – review & editing, Writing – original draft, Visualization, Supervision, Investigation, Conceptualization.

## Patient consent

Written consent to publish this case has not been obtained. This report does not contain any personal identifying information.

## Authorship

All authors attest that they meet the current ICMJE criteria for authorship.

## Funding

No funding or grant support.

## Declaration of competing interest

The authors declare the following financial interests/personal relationships which may be considered as potential competing interests:

Robert C Sergott reports a relationship with Heidelberg Engineering Inc that includes: consulting or advisory. Given Robert C Sergott's role as former Neuro-Ophthalmology section editor of American Journal of Ophthalmology Case Reports, had no involvement in the peer review of this article and had no access to information regarding its peer review. Full responsibility for the editorial process for this article was delegated to another journal editor. Daniel M Markowitz, Jirat Nimworaphan, Carl D Regillo, James P Dunn declare that they have no known competing financial interests or personal relationships that could have appeared to influence the work reported in this paper.
